# Amino-functionalized conjugated polymer electron transport layers enhance the UV-photostability of planar heterojunction perovskite solar cells†
†Electronic supplementary information (ESI) available: Experimental details, AFM, UPS. See DOI: 10.1039/c7sc00077d



**DOI:** 10.1039/c7sc00077d

**Published:** 2017-04-19

**Authors:** Dan Li, Chen Sun, Hao Li, Hui Shi, Xuxia Shai, Qiang Sun, Junbo Han, Yan Shen, Hin-Lap Yip, Fei Huang, Mingkui Wang

**Affiliations:** a Wuhan National Laboratory for Optoelectronics , Huazhong University of Science and Technology , Wuhan , Hubei 430074 , China . Email: mingkui.wang@mail.hust.edu.cn; b Institute of Polymer Optoelectronic Materials and Devices , State Key Laboratory of Luminescent Materials and Devices , South China University of Technology , Guangzhou , Guangdong 510640 , China . Email: msangusyip@scut.edu.cn ; Email: msfhuang@scut.edu.cn; c Wuhan National High Magnetic Field Center , Huazhong University of Science and Technology , 1037 Luoyu Road , Wuhan 430074 , Hubei , China

## Abstract

In this study, for the first time, we report a solution-processed amino-functionalized copolymer semiconductor (PFN-2TNDI) with a conjugated backbone composed of fluorine, naphthalene diimide, and thiophene spacers as the electron transporting layer (ETL) in n–i–p planar structured perovskite solar cells. Using this copolymer semiconductor in conjunction with a planar n–i–p heterojunction, we achieved an unprecedented efficiency of ∼16% under standard illumination test conditions. More importantly, the perovskite devices using this polymer ETL have shown good stability under constant ultra violet (UV) light soaking during 3000 h of accelerated tests. Various advanced spectroscopic characterizations, including ultra-fast spectroscopy, ultra-violet photoelectron spectroscopy and electronic impedance spectroscopy, elucidate that the interaction between the functional polymer ETL and the perovskite layer plays a critical role in trap passivation and thus, the device UV-photostability. We expect that these results will boost the development of low temperature solution-processed organic ETL materials, which is essential for the commercialization of high-performance and stable, flexible perovskite solar cells.

## Abstract 2

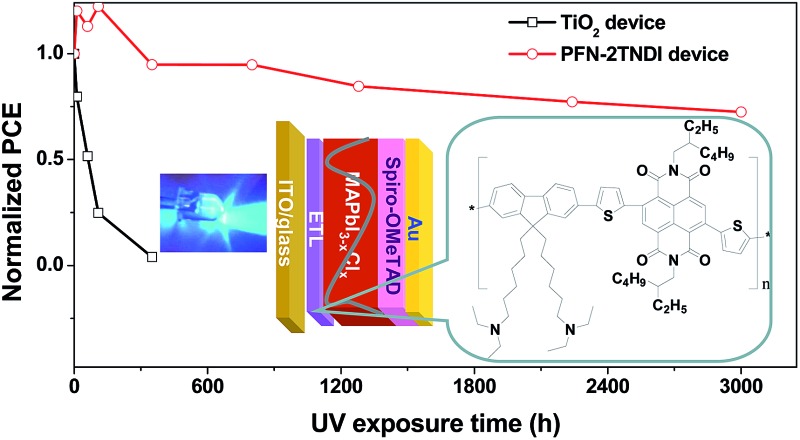
Long-term photostability and high performance were achieved by perovskite solar cells with an amino-functionalized conjugated polymer as a new electron transport layer.

## Introduction

As a new class of photovoltaic devices for solar energy utilization, perovskite solar cells have emerged as one of the promising alternatives to the conventional silicon-based photovoltaics over the past few years.^1–4^ These organic–inorganic hybrid lead halide perovskite materials are advantageous because they can be fabricated with solution-processable, relatively low cost methods, as compared to their silicon-based counterparts. The organic–inorganic hybrid perovskite materials possess superior optoelectronic properties, including high absorption coefficient (∼10^5^ cm^–1^),^5,6^ wide absorption (300–900 nm),^7,8^ small exciton binding energy (19–50 eV),^9^ long electron/hole diffusion length (100–1000 nm),^10,11^ suitable band gap (∼1.5 eV),^12^ and high bipolar conductivity (10^–2^–10^–3^ S cm^–1^).^13,14^ The efficiency of perovskite solar cells has increased from 3.8% in 2009 to 22.1% in 2016 through continuous efforts for the optimization of film deposition as well as device fabrication processes.^15^

Mesoporous- and planar-heterojunction (PHJ) structures are two main architectures adopted for efficient perovskite solar cells. In both device structures, the perovskite absorber layer is sandwiched between the electron transporting layer (ETL) and the hole transporting layer (HTL).^16,17^ Therefore, charge transporting layers are the key components of the perovskite devices, and they play an important role in improving both the efficiency and stability of the devices.^18,19^ The function of the charge transporting layer is to extract photon-generated charges from the perovskite layer and transport the charges to the corresponding current collecting electrodes. The dual crucial processes of fast charge transfer (forward) and slow recombination (backward) place challenging constraints on the choice of effective charge transporting layers. In general, a good charge transporting layer should have matched energy levels with the conduction band (CB) or valance band (VB) of the active layer, and should also possess high conductivity and charge mobility to ensure efficient charge transport, as well as good charge selectivity to increase the charge collection efficiency at the corresponding electrodes. Among the various materials, titanium dioxide (TiO_2_) is the most widely used ETL for electron transport and hole blocking, due to its good conductivity (1.1 × 10^–5^ S cm^–1^) and the suitable energy levels of the CB (∼–3.9 eV) and VB (∼–7.2 eV).^20–22^ Impressive PCEs over 19% have been obtained for PHJ perovskite solar cells using TiO_2_ as the ETL.^4,15^ However, the long-term operational stability of this type of device has been considered to be a major, and concern has been raised about its suitability for practical application. Most stability studies have so far focused on the moisture or heat effect, but some recent experimental results have indicated that the performance of n–i–p structure perovskite solar cells with TiO_2_ ETL suffer from rapid decay when exposed to illumination, even the devices that have been encapsulated in an inert atmosphere.^23–25^ Thus, there is an urgent need to investigate the corresponding degradation mechanism for this type of perovskite solar cells under light illumination and develop appropriate strategies to improve the long-term operational stability of the solar cells.

The application of these inorganic ETLs, particularly the crystalline TiO_2_, usually requires a high-temperature process to improve crystallinity and charge carrier mobility.^26,27^ High temperature sintering/annealing not only results in an increased cost and slow production, but also limits the utilization of plastic films as the flexible substrates. Therefore, replacing high-temperature processed ETLs with low-temperature processable materials can provide a better processing window and eventually simplify the manufacturing process of perovskite solar cells. Organic materials have several attractive features that can make them efficient ETLs, which can settle the challenges mentioned above. Recent studies have revealed that some organic ETLs could reduce the density of trap states on the surface and at the grain boundaries of perovskite crystals to improve the electron extraction efficiency and decrease photocurrent hysteresis.^28^ Despite many efficient organic ETLs being employed to improve the performance of p–i–n planar heterojunction perovskite solar cells, there are very few reports suggesting that organic ETLs could also be used in the n–i–p structure PHJ perovskite solar cells.^20,29,30^ Among them, fullerene (C_60_) and its derivative [6,6]-phenyl-C_61_-butyric acid methyl ester (PCBM) have been proved to be efficient ETLs in this type of perovskite solar cell with impressive PCEs (∼16%).^29,31^ Compared with these small molecular fullerenes, polymer semiconductors possess several appealing features that make them good candidates for ETL, such as good film formation properties, adjustable energy levels and excellent optical and electrical properties. Despite these advantages offered by polymer semiconductors, so far, they have not been explored as ETLs in the n–i–p structure PHJ perovskite solar cells.

**Fig. 1:**
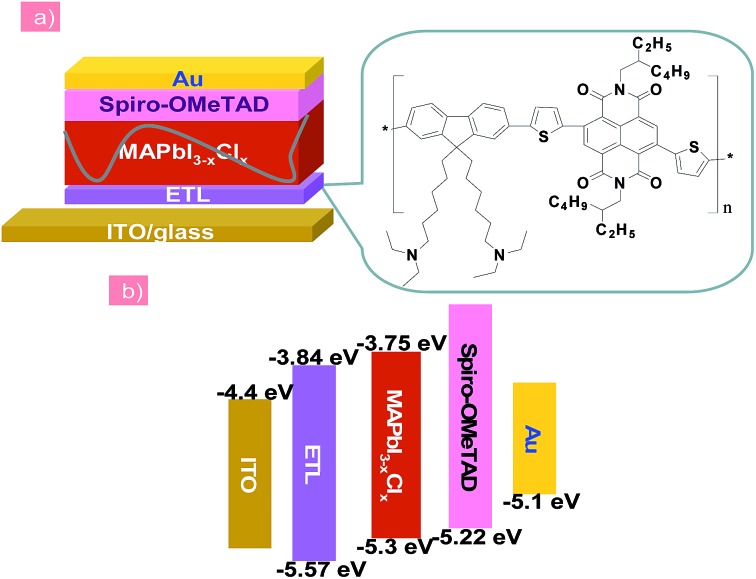
(a) The n–i–p structured planar heterojunction PSCs using the PFN-2TNDI electron transport layer. (b) Schematic energy band diagram of each layer.

In this work, we for the first time introduced an amino-functionalized copolymer as an alternative ETL to replace the commonly used TiO_2_ in the n–i–p PHJ perovskite solar cells with device configuration of ITO/ETL/MAPbI_3–*x*_Cl_*x*_/2,2′,7,7′-tetrakis-(*N*,*N*-di-*p*-methoxyphenylamine)-9,9′-spirobifluorene (spiro-MeOTAD)/Au, and studied the device stability under UV light soaking. Fig. 1 shows the chemical structure of the ETL material. The copolymer (named as PFN-2TNDI) has a conjugated backbone composed of fluorene, naphthalene diimide, and thiophene spacers and the synthetic routes are reported elsewhere.^32^ The matched energy levels, high electron mobility, and good film formation properties make it favorable as a good electron transporting material for perovskite solar cells. When it was used in a p–i–n planar perovskite solar cell with ITO/PEDOT:PSS/perovskite/ETL/metal electrode structure, the device showed a PCE of ∼16.7%.^32^ The introduction of PFN-2TNDI into n–i–p planar structure devices was inspired by a successful application of amino-substituted perylene diimide derivative (*N*-PDI) as a new generation ETL for this type of perovskite solar cell in our group.^20^ We found that the terminal amino groups in the *N*-PDI molecules could improve the wetting properties of the perovskite film, reduce the work function of the transparent conductive oxides substrate and also passivate the surface trap states of perovskite films. In this study, since the PFN-2TNDI also has amino groups in the alkylamine side chains, it may provide similar good properties for serving as an effective ETL. In addition, this ETL can be readily deposited to form a high-quality film due to the good film formation properties of the polymer, which significantly influence the morphology of the upper perovskite layer.^7,33^ Indeed, our study confirmed that the PFN-2TNDI could be used as an alternative ETL candidate for n–i–p perovskite solar cells with excellent performance. Devices based on the PFN-2TNDI ETL showed a competitive PCE of ∼16% under a simulated irradiation of AM 1.5G at 100 mW cm^–2^, which was comparable to the devices based on high-temperature sintered TiO_2_. More importantly, the devices based on PFN-2TNDI significantly inhibited UV induced performance degradation and exhibited excellent UV-photostability, which retained 75% of their original efficiency under constant UV exposure over 3000 hours; the control devices with TiO_2_ ETL only showed less than 10% of the original PCE value within 300 hours. This result highlights the possibility of using the low-temperature processed copolymer to replace inorganic metal oxide as ETLs for highly efficient and stable perovskite solar cells.

## Results and discussion

In this report, the n-type conjugated polymer PFN-2TNDI was used as the ETL in highly efficient n–i–p PHJ perovskite solar cells. Fig. 1a shows the device structure, in which we chose MAPbI_3–*x*_Cl_*x*_ as the absorber layer, PFN-2TNDI and spiro-OMeTAD as ETL and HTL, respectively. The MAPbI_3–*x*_Cl_*x*_ film was deposited on the substrate pre-coated with the PFN-2TNDI using a two-step inter-diffusion method.^34^ The spiro-OMeTAD HTL was deposited on top of the MAPbI_3–*x*_Cl_*x*_ layer by spin coating. The cells were finished by thermally evaporating an Au back electrode. Details of the fabrication processes are described in the Experimental section. As depicted in Fig. 1b, the energy levels of each layer are well-matched with the adjacent ones, which facilitate efficient electron/hole transport and extraction.

**Fig. 2:**
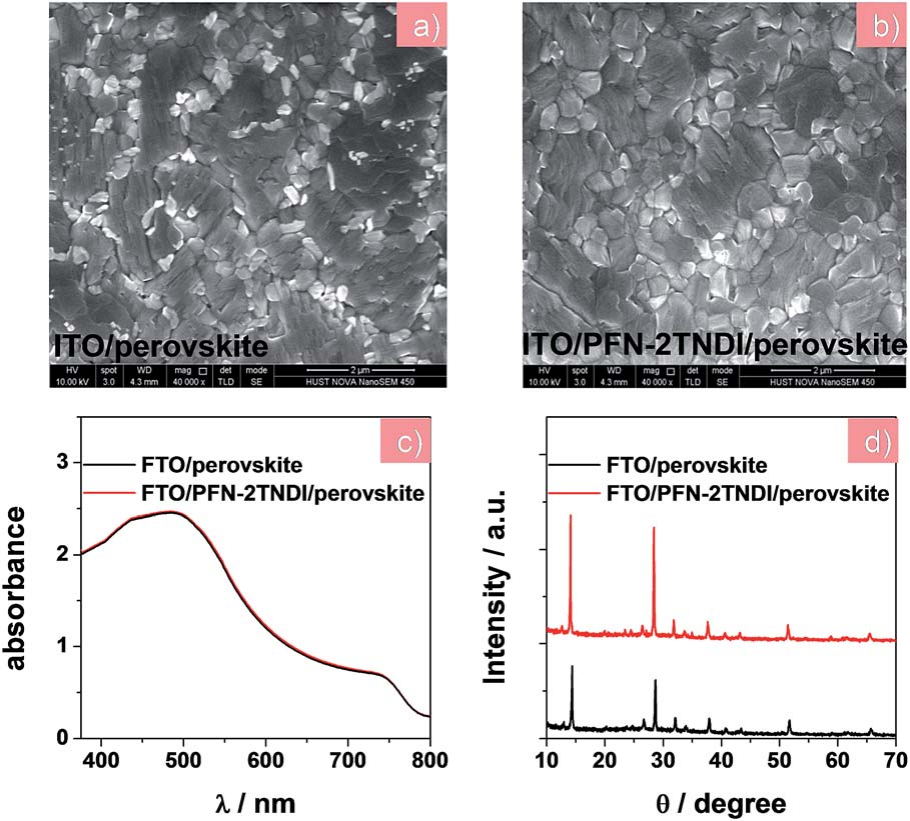
(a) and (b) SEM images, (c) UV-visible absorption spectra, (d) XRD patterns of MAPbI_3–*x*_Cl_*x*_ films on the ITO and ITO/PFN-2TNDI substrates, respectively.

Various characterizations, including top-view scanning electron microscopy (SEM), absorption spectra, and X-ray diffraction (XRD), were performed to illustrate the influence of the under-layered PFN-2TNDI on the structural properties of the MAPbI_3–*x*_Cl_*x*_ films. The SEM images in Fig. 2a and b show the surface morphology of the MAPbI_3–*x*_Cl_*x*_ films on ITO and PFN-2TNDI (5 nm) coated ITO substrate, respectively. Both perovskite films show compact multicrystalline structures with large grains (in the range of ∼1–2 μm), which is crucial for high-performance perovskite devices. Optical absorption spectra of both perovskite films on the ITO and ITO/PFN-2TNDI substrates (Fig. 2c) show broad absorption ranging from the visible to near-IR region. As we can see from the spectra, the absorption from the ultrathin ETL layers introduces ignorable changes in the absorption properties of the perovskites as a result of very intense light absorption properties of the thick perovskite films. The crystal structures of the MAPbI_3–*x*_Cl_*x*_ films were studied using XRD spectroscopy. As shown in Fig. 2d, the diffraction peaks of 14.2°, 28.4°, and 31.8° correspond to the (110), (220) and (310) planes of the tetragonal perovskite phase, confirming the formation of crystalline MAPbI_3–*x*_Cl_*x*_ in both films. However, the XRD intensity of the MAPbI_3–*x*_Cl_*x*_ film deposited on the ITO/PFN-2TNDI substrate was much stronger than that deposited on the bare ITO substrate, indicating that the ETL promoted the formation of a perovskite with higher phase purity and preferential orientation, which are important factors for high performance devices.

**Fig. 3:**
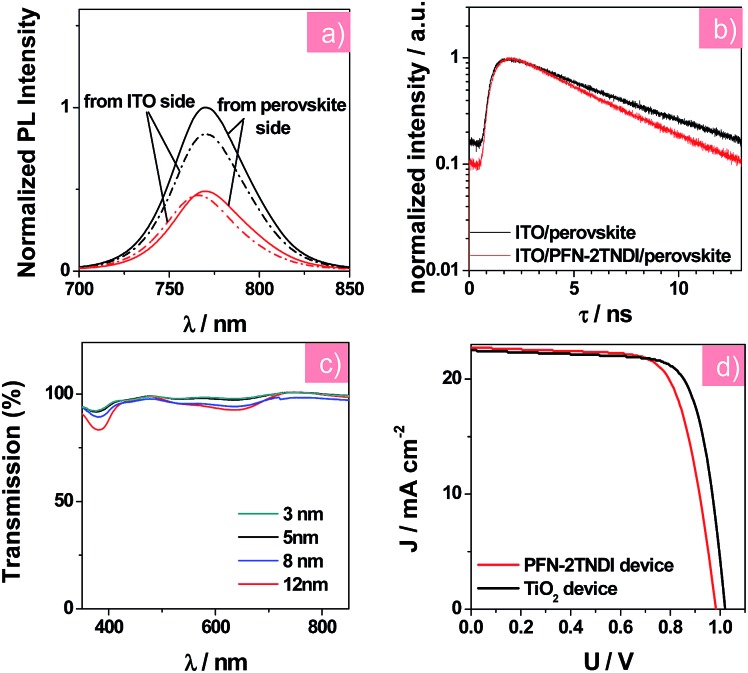
(a) The steady-state PL spectra of MAPbI_3–*x*_Cl_*x*_ film on different substrates with an excitation wavelength of 400 nm from the ITO side and the perovskite side; red curves represent the ITO/PFN-2TNDI substrate, black curves represent the ITO substrate. (b) Time-resolved PL decay of MAPbI_3–*x*_Cl_*x*_ films on the ITO and ITO/PFN-2TNDI. (c) Transmission spectra of PFN-2TNDI films with different thicknesses after extraction by ITO. (d) *J*–*V* curves of solar cells based on the PFN-2TNDI (5 nm), and TiO_2_. The applied voltage scan rate in *J*–*V* measurement was kept at the same 10 mV per step with a delay time of 5 ms in the reverse scan direction.

Steady-state photoluminescence (PL) and time-resolved PL decay measurements were further conducted to investigate the charge transfer and recombination occurring at the perovskite/ETL interface. According to earlier reports,^20,32^ the amino groups of this copolymer's side chains could passivate the surface trap states, which were introduced by halide vacancies in MAPbI_3–*x*_Cl_*x*_ films, leading to a recovery of the bandgap. The MAPbI_3–*x*_Cl_*x*_ film deposited on the ITO/PFN-2TNDI substrate shows stronger PL quenching than that on the bare ITO substrate (as in Fig. 3a), suggesting that the PFN-2TNDI is a good quencher for the perovskite films. An excitation wavelength of 400 nm was used to excite the perovskite films on the PFN-2TNDI modified substrates from either the ITO side or the perovskite side to investigate the trap passivation effect at the ETL/perovskite interface. Moreover, the perovskite film with PFN-2TNDI shows a blue-shifted PL peak from 769 (excited from the perovskite side) to 764 nm (excited from the ITO side), indicating a signature of filling the trap states close to the bottom surface of the MAPbI_3–*x*_Cl_*x*_ film by the PFN-2TNDI.^28,32^ In contrast, the perovskite film coated on the bare ITO maintains its PL peak at 769 nm under excitation from both sides. One potential explanation for the surface trap passivation is that the electron rich nitrogen atoms on the alkylamine side chain of PFN-2TNDI may coordinate with the unsaturated Pb atoms at the MAPbI_3–*x*_Cl_*x*_ surface, thus providing an effective trap passivation effect at the ETL/perovskite interface. Furthermore, the perovskite film on the ITO/PFN-2TNDI substrate underwent a faster PL decay as described in Fig. 3b. The PL decay curves were fitted with a single-exponential equation with PL lifetime values of 5.56 and 4.59 ns for the MAPbI_3–*x*_Cl_*x*_ films on ITO and ITO/PFN-2TNDI substrates, respectively. The shorter lifetime for the PFN-2TNDI device suggests that a faster carrier extraction can be achieved from the MAPbI_3–*x*_Cl_*x*_ film to the ETL, further demonstrating that PFN-2TNDI is an efficient ETL.

The film formation properties of PFN-2TNDI were studied using atomic force microscopy (AFM). As illustrated in the AFM topography images (Fig. S1, ESI†), the root mean square (RMS) roughness value of the quartz substrate was reduced from 1.189 to 0.328 nm by coating with 5 nm-thick PFN-2TNDI, suggesting that this polymer has good film formation properties. The smooth PFN-2TNDI surface facilitates the formation of perovskite films with higher surface coverage and reduced pinhole size.^7,33^ Ultraviolet photoelectron spectroscopy (UPS) measurement was conducted to study the change in the work function (WF) of the ITO surface when coated with the ETL. We found that the WF of the ITO was reduced to 3.81 eV from its original 4.4 eV after the deposition of PFN-2TNDI (5 nm), as displayed in Fig. S2,† which was a little higher than that of the TiO_2_-coated ITO sample (3.76 eV). This value matches well with the CB of MAPbI_3–*x*_Cl_*x*_ film, which facilitates ohmic contact formation and efficient charge transfer at this interface. The reduction of the substrate's WF can be attributed to a large dipole moment at the ITO/PFN-2TNDI interface, induced by the amino groups on the PFN-2TNDI side chain, which has been widely discussed in the field of polymer solar cells.^20^ The chemical properties of PFN-2TNDI are different from those of TiO_2_, which may affect the formation of perovskite crystals on them. Therefore, we compared the wetting capability of PFN-2TNDI and TiO_2_ substrate surfaces (Fig. S3†). The average contact angles are 30° and 100° for the PFN-2TNDI and TiO_2_ films to water, respectively. Because of the similar polarity of *N*,*N*-dimethylformamide (DMF) to water, we expect that this is an indication of the wettability of these surfaces by the perovskite precursor. It was reported that when the two-step inter-diffusion method was employed, the hydrophobic surfaces were good for the formation of perovskite films with large crystalline grains and less charge trap density by preventing the formation of too dense nuclei from heterogeneous nucleation.^35^

We carried out systematic characterizations to find out the optimum thickness of PFN-2TNDI as the ETL in the MAPbI_3–*x*_Cl_*x*_ solar cells. Fig. 3c shows the transmission spectra of ITO/PFN-2TNDI samples obtained by spin-coating PFN-2TNDI from solutions of different concentrations. The transmittance of the samples gradually decreased as the film thickness of PFN-2TNDI increased from 3 nm to 12 nm, especially in the range of 350–450 nm and 560–700 nm, which can be attributed to the absorption from the π–π* transition and intra-molecular charge transfer characteristics of the PFN-2TNDI film.^32^ For comparison, Fig. S4† shows the transmission spectra of the TiO_2_ compact layer. The PFN-2TNDI film with a thickness of 5 nm showed slightly less transmittance than the TiO_2_ film in the short wavelength range. This ensures the absorption of the perovskite layer deposited above it (Fig. 2c). The photovoltaic performances of the perovskite solar cells with a configuration of ITO/PFN-2TNDI/MAPbI_3–*x*_Cl_*x*_/spiro-OMeTAD/Au were evaluated by varying the PFN-2TNDI film thicknesses. A control device without the PFN-2TNDI was also presented. Table 1 lists the photovoltaic parameters for various perovskite devices under the illumination of AM 1.5G, 100 mW cm^–2^. As can be seen, the device performances significantly depend on the thickness of the PFN-2TNDI films. The optimized thickness of the PFN-2TNDI is about 5 nm. Further increasing the thickness results in the deterioration of the device performance due to an increase in electrical resistance (Table 1). Perovskite solar cells with an optimized PFN-2TNDI film exhibit a short-circuit current density (*J*_SC_) of 22.01 mA cm^–2^, a fill factor (FF) of 0.74, an open-circuit voltage (*V*_OC_) of 0.98 V, yielding an impressive PCE value of 15.96% (Table 1). The control device without ETL achieves a PCE of 11.99% with *V*_OC_, *J*_SC_ and FF of 0.91 V, 19.42 mA cm^–2^ and 0.68, respectively. The enhancement of *J*_SC_ and FF of the device with PFN-2TNDI ETL could be partially attributed to the passivation effect of PFN-2TNDI on the defects in perovskite films, which reduces the charge recombination at the interface.^32,36^ Additionally, the rectification effect (*i.e.*, efficient electron extraction and hole blocking) induced by the PFN-2TNDI interlayer could lead to improvement in *J*_SC_ and FF. The enhanced *V*_OC_ from 0.91 V of the control device to 0.98 V of the PFN-2TNDI-based device can be attributed to better energy alignment at the ITO/ETL and ETL/perovskite interfaces.^20,32^ Therefore, the existence of the PFN-2TNDI layer minimizes the potential loss between the ITO and perovskite and enlarges the built-in potential across the device.^37,38^ Consequently, the enlarged built-in field could reduce carrier accumulation and further reduce carrier recombination inside the solar cell.

**Table 1:** Photovoltaic parameters of the perovskite solar cells on different substrates under illumination of AM 1.5G, 100 mW cm^–2^

Samples		*V*_OC_ (V)	*J*_SC_ (mA cm^–2^)	FF	PCE (%)	*R*_s_ (Ω cm^2^)
ITO/No. ETL		0.91	19.42	0.68	11.99	3.75
ITO/PFN-2TNDI (*x* nm)	3	0.91	21.89	0.70	14.04	3.48
	5	0.98	22.01	0.74	15.96	2.16
	8	0.95	20.25	0.70	13.56	3.85
	12	0.96	19.68	0.68	12.89	4.34
ITO/TiO_2_		1.00	21.66	0.78	17.2	1.86

We further compared the PFN-2TNDI-based devices with the TiO_2_-based ones to evaluate the viability of PFN-2TNDI as ETL in perovskite solar cells. Fig. 3d compares the *J*–*V* curves of solar cells based on the PFN-2TNDI (5 nm) and TiO_2_. The corresponding photovoltaic parameters for the TiO_2_ (30 nm)-based devices are also shown in Table 1. With an optimized TiO_2_ thickness of ∼30 nm, the TiO_2_-based device exhibited a PCE of 17.2% with a *V*_OC_ of 1.00 V, a *J*_SC_ of 21.66 mA cm^–2^ and a FF of 0.78. Impressively, the optimized performances of devices based on PFN-2TNDI ETL (5 nm) are comparable to that of TiO_2_-based devices, clearly indicating that the copolymer PFN-2TNDI can be used as an outstanding solution-processed ETL for perovskite devices. The relatively higher *J*_SC_ of PFN-2TNDI-based devices could be ascribed to the larger perovskite crystalline grains on PFN-2TNDI, which led to an increase in IPCE value at longer wavelength (Fig. S5†). The delicate difference in *V*_OC_ between PFN-2TNDI-based devices and TiO_2_-based devices may be caused by the different work functions of PFN-2TNDI and TiO_2_.

**Fig. 4:**
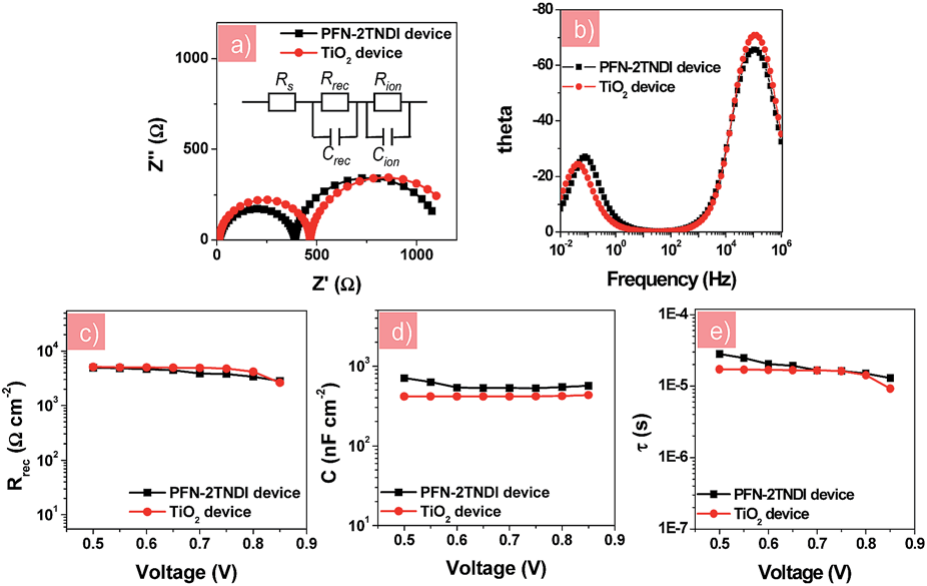
(a) Nyquist plots and (b) Bode plots of perovskite solar cells with different electron transport layers; (c) the recombination resistance *R*_rec_; (d) the capacitance (*C*) and (e) recombination lifetime (*τ*) derived from the high frequency region as a function of the applied voltage.

In order to further understand the electronic transport processes at the perovskite/ETLs interfaces, electronic impedance spectroscopy (IS) measurement was performed for the n–i–p perovskite solar cells based on low-temperature processed PFN-2TNDI and high-temperature processed TiO_2_. Fig. 4a and b show the Nyquist plots and Bode plots for both devices under illumination at a bias of –0.7 V in the frequency range of 1 MHz–10 mHz, from which two semicircles could be easily identified. The results indicate that similar interfacial charge transfer processes occur in both devices based on PFN-2TNDI and TiO_2_. Herein, the semicircle in the range of high frequency provides useful information on the charge carrier recombination process, *i.e.*, interfacial recombination of electrons from the ETL with holes from MAPbI_3–*x*_Cl_*x*_, or electrons from MAPbI_3–*x*_Cl_*x*_ with holes from HTL^39,40^ and the charge transfer process at the ITO and Au electrodes.^16,40^ The latter is too fast to be separated from the former. The semicircle in the relatively low frequency represents ion migration processes in the perovskite active layer.^16,33^ The Nyquist plots were fitted using an equivalent circuit model of two-RC elements in series as shown in Fig. 4a. For a better fitting, all capacitor elements were replaced by constant phase elements; in all the cases, the constant phase element (CPE) exponent *p* was kept quite closely to the perfect capacitor value, *p* ≈ 1. Fig. 4c and d present the charge recombination resistance (*R*_rec_) and geometrical capacitance (*C*) as a function of bias for devices based on PFN-2TNDI and TiO_2_ ETL. It is interesting to find that the recombination resistance (*R*_rec_, Fig. 4c) in the PFN-2TNDI-based device is almost the same as that of the TiO_2_-based device. These results suggest that the charge flux for recombination processes in both devices are within the same order. However, the utilization of PFN-2TNDI shows larger capacitance (*C*, Fig. 4d), indicating that more charge would take part in the interfacial recombination process. This conclusion is based on the density of states, DOS, which can be reflected by capacitance through the approximation of DOS ∼ *C*. This explains why the device using PFN-2TNDI shows lower output photo-voltage than that of the TiO_2_-based device (Table 1), even though a longer electron lifetime is observed in the former case (Fig. 4e). The IS results further confirm that the PFN-2TNDI can serve as an efficient ETL, like the TiO_2_, and provide an alternative choice for fabrication of n–i–p PHJ perovskite solar cells.

**Fig. 5:**
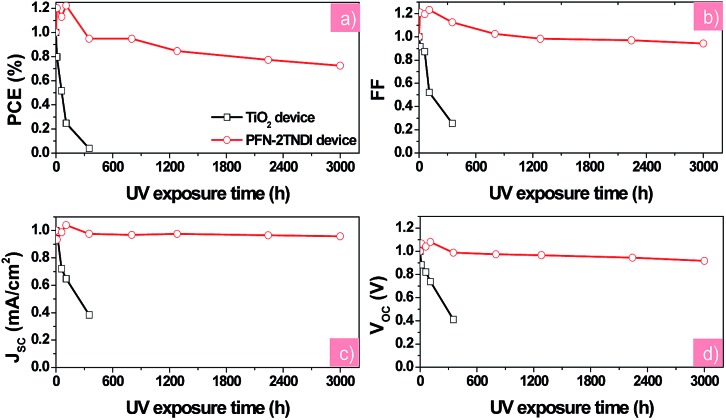
The evolution of solar cell parameters (normalized (a): *V*_OC_, (b): *J*_SC_, (c): FF, (d): PCE) as a function of testing time under UV illumination for the PFN-2TNDI- and TiO_2_-based perovskite solar cells. The devices were stored under exposure to UV in a N_2_-filled glove-box during testing.

Stability measurements were also performed for the two devices under constant UV illumination. Fig. 5 presents the evolution of solar cell performance parameters as a function of testing time under UV illumination of the PFN-2TNDI- and TiO_2_-based perovskite solar cells. Those devices were encapsulated with glass cover-slips glued with epoxy resin. The devices were exposed to constant 365 nm UV light through ITO sides in an argon-filled glove-box with a humidity of <1.0 ppm. We found that all the photovoltaic parameters for the PFN-2TNDI-based perovskite solar cells improved a little within the first 20 h of illumination and then slowly degraded to about 75% over the next period of aging time (3000 hours) with small variation in *V*_OC_, *J*_SC_, and FF. In contrast, for the TiO_2_-based devices, the PCE dropped significantly to less than 10% of the original value within 300 hours of illumination, with a particularly rapid degradation in *V*_OC_, *J*_SC_, and FF. These results suggest that the perovskite solar cells based on PFN-2TNDI ETL are much more stable than the TiO_2_ analogues under UV irradiation. Since the difference in the devices is related to ETL, we further conclude that the light-activated degradation at the interface between TiO_2_ and perovskite is the major reason accounting for the degradation of the devices. In addition to the essential characteristics of an n-type semiconductor, the excited TiO_2_ has a strong ability to extract electrons from electron-rich materials. Therefore the TiO_2_ has been used as a typical photocatalyst for environmental purification, such as CO_2_ reduction, organic compounds decomposition and water splitting.^41–44^ Hence, electron extraction from the iodide anion in MAPbI_3–*x*_Cl_*x*_ by the TiO_2_ may provide a driving force for the deconstruction of the photoactive layer, resulting in the deterioration of device performance.^24^ Other reports suggest that the UV degradation of perovskite solar cells originates from a large amount of oxygen vacancies in the TiO_2_ layer.^23^ Upon excitation by UV light, these oxygen vacancies could act as deep electron trap states. The photo-generated electrons by the MAPbI_3–*x*_Cl_*x*_ layer could be trapped by these defeat states and then recombine with holes, and thus reduce the charge collection efficiency and cause further deterioration of the device performance. However, the organic PFN-2TNDI has less UV-light-induced structural defects and traps. Consequently the devices based on PFN-2TNDI have indeed shown much improved resilience to UV irradiation. A little deterioration observed with this device would be caused by the de-doping of spiro-OMeTAD and the reaction between spiro-OMeTAD and the Au electrode.

**Fig. 6:**
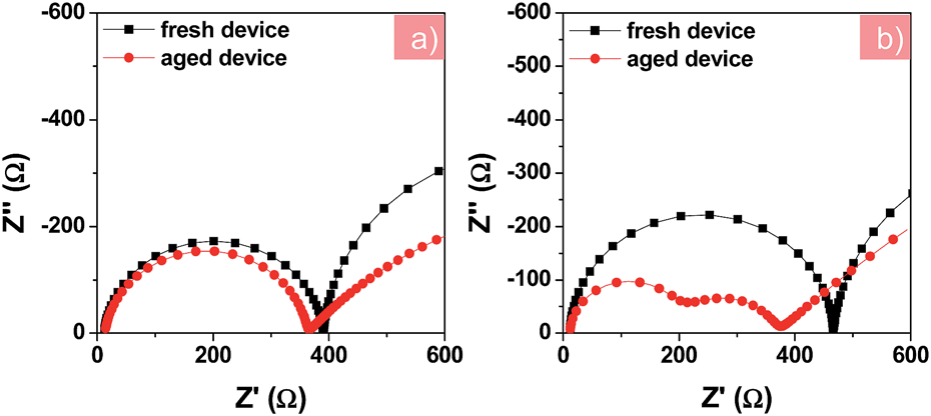
Nyquist plots of fresh and aged devices based on PFN-2TNDI (a) and TiO_2_ (b) under illumination at a bias of –0.7 V. The aged devices were under exposure to constant UV light in an argon-filled glove box for 200 h before testing.

To further understand the change in electronic processes in the PFN-2TNDI- and TiO_2_-based devices after the UV irradiation, we further analyzed their Nyquist plots as shown in Fig. 6a and b, respectively. The fresh and aged devices using the PFN-2TNDI ETL show two similar semicircles in the Nyquist plots, in which only a small decrease can be found in the first semi-arc in the high frequency range, suggesting that there is no significant change in the interfacial electronic processes in this type of device after exposure to UV-light. In contrast, an additional semi-arc appears for the aged devices with the TiO_2_ ETL in the high frequency range. The newly emerged semicircle implies that an additional interfacial process is generated during the UV aging process, which might adversely affect the carrier transport in the devices.^31^ A large reduction in the recombination resistance (*R*_rec_) was observed in the TiO_2_-based device after UV-exposure, probably due to an increase in charge recombination at the MAPbI_3–*x*_Cl_*x*_/TiO_2_ interface. This led to the dramatically fast degradation of the device performance, especially the *V*_OC_ and FF (Fig. 5). Interestingly, the UV-light aging process showed a less significant effect on the Au/HTM interface for both devices, according to the IS results shown in Fig. 6.

## Experimental

The etched ITO (indium tin oxide) substrates were ultrasonically cleaned in detergent, milli-Q water, acetone and ethanol for 15 min, respectively. Prior to depositing the ETLs, the clean and dry ITO substrate was treated with UV/O_3_ for 30 min. PFN-2TNDI layers were coated on the ITO by spin coating at 3000 rpm for 30 s using a solution of PFN-2TNDI in chlorobenzene with various concentrations of 0.5 mg mL^–1^, 1 mg mL^–1^, 2 mg mL^–1^ and 4 mg mL^–1^, then annealed at 100 °C for 10 min. The MAPbI_3–*x*_Cl_*x*_ film was fabricated by a two-step inter-diffusion method.^34^ The PbI_2_ layer was spin coated from the 70 °C pre-heated PbI_2_ solution in DMF (462 mg mL^–1^), then 30 μL of the pre-heated solution was dropped onto the ETL-covered ITO substrate as soon as possible, and then the spinning was immediately started at 3000 rpm. The PbI_2_ film was annealed at 70 °C for 30 min. After cooling to room temperature, 25 μL MAI : MACl (50 : 5 mg) in 1 mL 2-propanol was dropped on the as-fabricated PbI_2_ film and spin coating was taken at 3000 rpm for 30 s. After the deposition of stacked precursor layers, the obtained films were annealed on the hotplate at 135 °C for 15 min. The HTL was deposited on top of perovskite film by spin coating at 4000 rpm for 30 s. The acceleration was 3000 rpm per second. The spin coating solution was composed of 72.3 mg spiro-OMeTAD in 1 mL chlorobenzene with the standard additives of lithium bis(trifluoromethylsulphonyl)imide in acetonitrile of 520 mg mL^–1^ (17.5 μL) and 4-*tert*-butylpyridine (30 μL). Then, an 80 nm-thick gold electrode was thermally evaporated under the vacuum pressure of 5.0 × 10^–4^ Pa to complete the device fabrication. All the spin-coating processes were performed in a dry air-filled glove box with the humidity of <1.0 ppm. The effective area of the solar cell was defined to be 0.125 cm^2^. For the control device based on the TiO_2_, all the fabrication processes were the same, except that the compact layer was produced by spin coating at 3000 rpm twice using nanocrystalline TiO_2_ solution precursors, and then annealed on a hot plate for 30 min at 150 °C. The synthesis methods of TiO_2_ precursors are available in the ESI.†

Other experimental details including materials and device characterization are shown in the ESI.†

## Conclusions

In summary, a polymer semiconductor PFN-2TNDI has been demonstrated as an efficient ETL for n–i–p structured perovskite solar cells. The perovskite devices incorporated with the PFN-2TNDI ETL showed good performance with PCE of ∼16%, which was comparable to that of inorganic TiO_2_ ETL-based devices. More importantly, in addition to the low temperature processability offered by the polymer ETL, the devices based on the polymer ETL show greatly enhanced photostability against UV irradiation, compared to the TiO_2_-based devices. We believe this work not only can provide important insights on designing new organic materials as efficient interfacial layers for high performance perovskite solar cells, but also demonstrates a feasible strategy to overcome the photostability issue encountered in perovskite devices using inorganic semiconductors as the charge transport layer.
