# Supplementary material for: Amino-functionalized conjugated polymer electron transport layers enhance the UV-photostability of planar heterojunction perovskite solar cells
Source: Chem Sci. 2017 Apr 19;8(6):4587–94. doi: 10.1039/c7sc00077d (PMC5618109; doi:10.1039/c7sc00077d)
Supplement: Supplementary file 1 [file SC-008-C7SC00077D-s001.pdf]

Electronic Supplementary Material (ESI) for Chemical Science.
This journal is © The Royal Society of Chemistry 2017

Supporting Information

Amino-Functionalized Conjugated Polymer Electron Transport layers Enhances UV-Photostability of Planar Heterojunction Perovskite Solar Cells

Dan Li [a], Chen Sun [b], Hao Li [a], Hui Shi [b], Xuxia Shai [a], Qiang Sun [a], Junbo Han [c], Yan Shen [a], Hin-Lap Yip [b], Fei Huang [b]* and Mingkui Wang [a]**

[a] Wuhan National Laboratory for Optoelectronics, Huazhong University of Science and Technology, Wuhan, Hubei 430074, China.

[b] Institute of Polymer Optoelectronic Materials and Devices, State Key Laboratory of Luminescent Materials and Devices, South China University of Technology, Guangzhou, Guangdong 510640, China.

[c] Wuhan National High Magnetic Field Center, Huazhong University of Science and Technology, 1037 Luoyu Road, Wuhan 430074, Hubei, China

* Corresponding authors. E-mail: mingkui.wang@mail.hust.edu.cn (M.W.); E-mail: msangusyip@scut.edu.cn (H.Y.); E-mail: msfhuang@scut.edu.cn (F.H.)

1. Materials

Methylammonium iodide (MAI) and methylammonium chloride (MACl) were synthesized according to literature.[33] Briefly MAI (or MACl) was synthesized by adding methylamine (40% in methanol, Aladdin) and hydroiodic acid (57% in water, Aldrich) (or hydrochloric acid, 37% in water) in 1:1 molar ratio into a beaker at 0 °C with stirring for 2 hours. After reaction, the solvents were removed by rotary evaporation and the products were recrystallized and washed by hexane several times to get white crystals. The TiO_2 nanoparticles were synthesized according to the following steps. Firstly, 0.5 mL of $TiCl_4$ (Aldrich, 99.8%) was added into 2 mL of ethanol and then mixed in 10 mL of anhydrous benzyl (Aldrich, 99.8%), obtaining a yellow solution. Then, the solution was heated at 80° C for 9 h. 1 mL volume of the resulting suspension was precipitated in 12 mL of diethyl ether and centrifuged at 4500 rpm to isolate the nanoparticles. The synthesized nanoparticles were dispersed in ethanol, receiving a transparent solution of nanocrystals with a concentration of 5.3 mg mL^{-1}. Lead iodide (PbI_2, 99%) was purchased from Sigma-Aldrich. 2,2',7,7'-tetrakis-(N,N-di-pmethoxyphenylamine)-9,9'-spirobifluorene (spiro-OMeTAD) was purchased from Luminescence Technology Corp. All the chemicals were used as received without further purification.

2. Sample characterization

UV-visible absorption of the $MAPbI_{3-x}Cl_x$ films deposited on different substrates was measured to understand the effect of PFN-2TNDI on the perovskite optical property using UV 3600 spectrophotometer (SHIMADZU). Other characteristics such

as atomic force microscopy (AFM) were employed to provide a better understanding of morphology of the PFN-2TNDI films. The surface roughness of substrates coated with or without PFN-2TNDI was also measured by AFM (SPM9700, Shimadzu). The work function of electrodes plays a critical role in charge extraction in PHJ perovskite solar cells. Therefore, ultraviolet photoelectron spectroscopy study (UPS, AXIS-ULTRA DLD-600W) was performed to further investigate the electronic effect of the PFN-2TNDI film on the work function of ITO. Current-voltage (J-V) characteristics were recorded on the Keithley model 2400 digital source meter (USA) under the AM 1.5G illumination condition. A solar simulator consisting of a Xe light source (450 W, Oriel, model 9119) and an AM 1.5G filter (Oriel, model 91192) was used to produce an irradiance of 100 mW cm^{-2} on the surface of the solar cells. The intensity of simulated light was calibrated with an NREL certified KG5 filtered Si reference diode. The J-V data was acquired with reversed scan in the potential range from 1.2 V to -0.2 V with a scan rate of 10 mV s^{-1}. X-ray diffraction (XRD) measurements were performed by Philips X-ray diffractometer with Cu Kα radiation. The samples were scanned from 10° to 80° with a step-size of 0.02°. The top-view scanning electron microscopy (SEM) images were taken by Nano SEM 450 (Tecnai G20). Time-resolved photoluminescence (PL) decay spectrum was measured using time correlated single photo counting system (PicoHarp 300, PicoQuant GmbH) with an excitation laser beam at 400 nm. Electronic impedance spectra (EIS) measurements were taken on an Autolab Frequency Analyzer setup, which consists of the Autolab PGSTAT 30 (Eco Chemie B.V., Utrecht, The Netherlands) and the Frequency Response Analyzer

module, in the frequency range from 1 MHz to 0.01 Hz with oscillating amplitude of 30 mV. Z-view software (v2.8b, Scribner Associates Inc.) was used to fit the obtained impedance spectra.

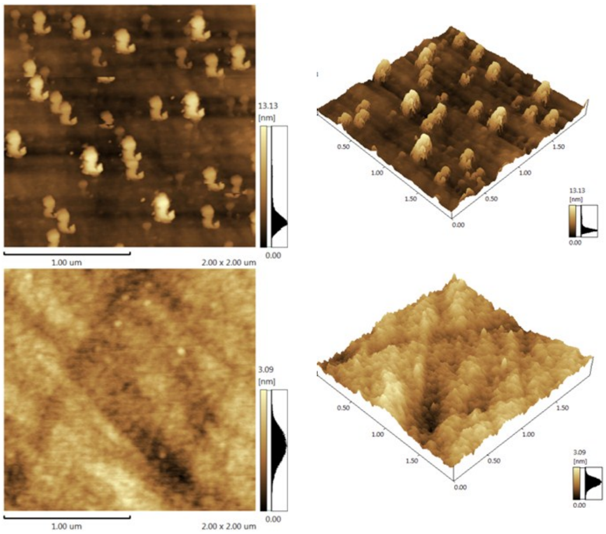

Fig. S1 Three-dimensional AFM images and topography of quartz substrates with coated with (above) and without (below) PFN-2TNDI.

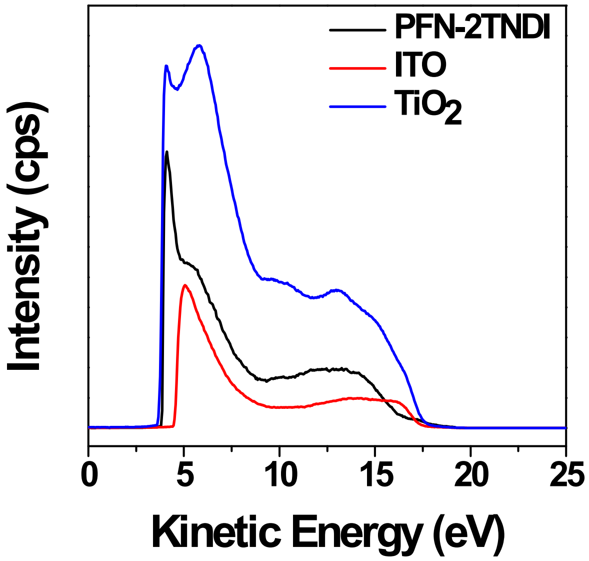

Fig. S2 UPS measurements for ITO, PFN-2TNDI and TiO_2.

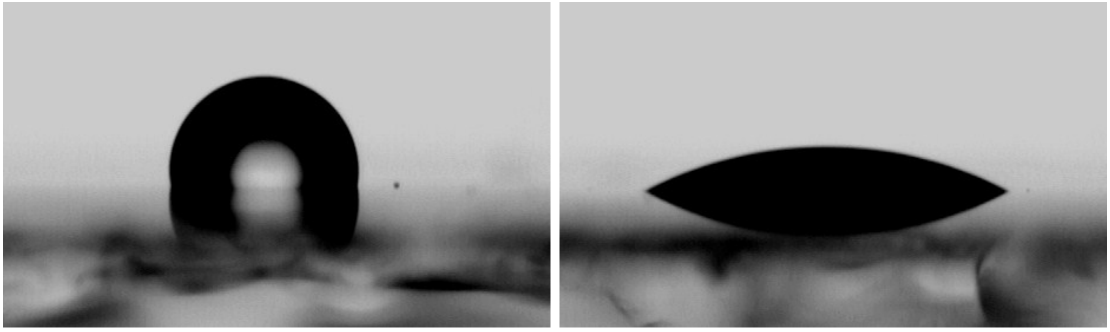

Fig. S3 The contact angle of water on the PFN-2TNDI (left) and TiO_2 (right) substrate surfaces.

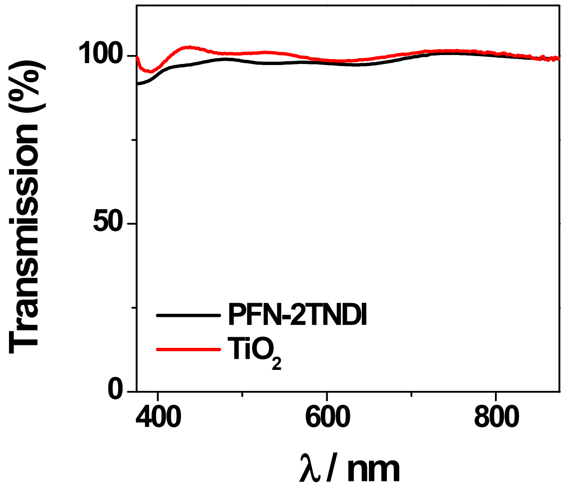

Fig. S4 Transmission spectrum of PFN-2TNDI (5nm) and TiO_2 films after extraction by ITO substrates.

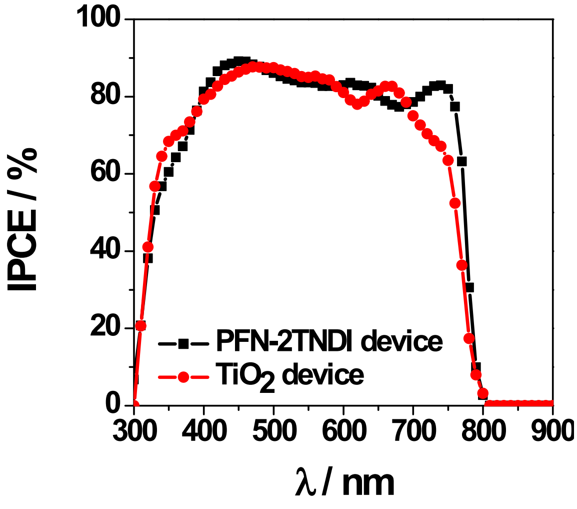

Fig. S5 IPCE of the devices based on PFN-2TNDI ETL and TiO_2 ETL.
